# Gravity as a Strong Prior: Implications for Perception and Action

**DOI:** 10.3389/fnhum.2017.00203

**Published:** 2017-04-28

**Authors:** Björn Jörges, Joan López-Moliner

**Affiliations:** ^1^Department of Cognition, Development and Psychology of Education, Faculty of Psychology, Universitat de BarcelonaCatalonia, Spain; ^2^Institut de Neurociències, Universitat de BarcelonaCatalonia, Spain

**Keywords:** gravity perception, perceptual adaptation, bayesian framework, strong prior, optic flow, catching, perception and action

## Abstract

In the future, humans are likely to be exposed to environments with altered gravity conditions, be it only visually (Virtual and Augmented Reality), or visually and bodily (space travel). As visually and bodily perceived gravity as well as an interiorized representation of earth gravity are involved in a series of tasks, such as catching, grasping, body orientation estimation and spatial inferences, humans will need to adapt to these new gravity conditions. Performance under earth gravity discrepant conditions has been shown to be relatively poor, and few studies conducted in gravity adaptation are rather discouraging. Especially in VR on earth, conflicts between bodily and visual gravity cues seem to make a full adaptation to visually perceived earth-discrepant gravities nearly impossible, and even in space, when visual and bodily cues are congruent, adaptation is extremely slow. We invoke a Bayesian framework for gravity related perceptual processes, in which earth gravity holds the status of a so called “strong prior”. As other strong priors, the gravity prior has developed through years and years of experience in an earth gravity environment. For this reason, the reliability of this representation is extremely high and overrules any sensory information to its contrary. While also other factors such as the multisensory nature of gravity perception need to be taken into account, we present the strong prior account as a unifying explanation for empirical results in gravity perception and adaptation to earth-discrepant gravities.

## Introduction

Internally represented and/or perceived gravity has been shown to play a role in sensorimotor tasks, such as catching (Lacquaniti and Maioli, 1989b; Tresilian, 1993; de la Malla and López-Moliner, 2015), motor control (Bock et al., 1992; Gaveau et al., 2011), spatial perception (Clément et al., 2008, 2013) and even for the perception of movement patterns (Westhoff and Troje, 2007; Maffei et al., 2015). Furthermore, especially when it comes to tasks like catching or trajectory estimation, humans are exceptionally adapted to the earth gravity value of 9.81 m/s^2^: random accelerations are hardly perceived at all (Werkhoven et al., 1992) and generally impair catching behavior (Brenner et al., 2016). Nonetheless, we catch objects that accelerate downwards according to earth gravity just fine (Zago et al., 2005), even when parts of their trajectory is occluded and we thus do not have any cues on position and velocity during flight (Lacquaniti and Maioli, 1989a). What is more, humans expect descending objects to accelerate even when their velocity is constant (Zago et al., 2004). While details will be discussed later, it is safe to say that humans are highly attuned to earth gravity conditions and that it is very likely that any earth-discrepant gravity conditions pose a big challenge to the perceptual system.

There are several reasons why this concern is relevant: space travel has come a long way since, in 1957, Sputnik 2 with mongrel Laika on board brought the first living being to space. Humans walked the surface of the Moon and spent large periods in space, like Russian Valeri Polyakov with 437.7 days, or most recently Scott Kelly with 340 days in preparation of a manned mission to Mars. While physiological effects like muscle atrophy have received a lot of attention (Vandenburgh et al., 1999; Fitts et al., 2001), the influence of prolonged stays in space on perceptual functions remains largely unexplored. Research on gravity can not only provide us with a better understanding of the challenges that astronauts face on their missions to space, but also can it give us an idea of what it would be like for humans to live in space for prolonged time spans. But space is not the only place where we might be exposed to altered gravity conditions: with virtual environments being used for a multitude of purposes, from research and training simulations to recreational applications, its possibilities become more and more apparent. It delivers a world in which stimuli can be controlled in every possible way, including the laws of physics, at least as far as their visual presentation is concerned. Manipulation of gravity can have different purposes: altered visual gravity has been used in training protocols for gait and balance problems (Oddsson et al., 2007) and it serves as an opportunity to practice complex tasks for space missions (Bruyns et al., 2001); and then there is a whole range of business and leisure activities—grouped together under the umbrella of “Augmented Reality”—that will eventually come around playing with different gravity values. Research in the area will thus shed light on what kind of constraints there are for the construction of virtual reality environments in terms of gravity and how strictly they need to be adhered to in order to guarantee a positive user experience. Finally, it will also grant insights into how conflicts between visual and bodily (vestibular, proprioceptive etc.) gravity cues are negotiated.

To cut a long story short: technology is ramping up and humans will inevitably be exposed to non-earth gravity conditions for increased durations—in space or in virtual reality–, be it for scientific purposes, as a remedy for a growing earth population or simply for the fun of it. On the other hand, as the human perceptual system is highly attuned to earth gravity, these technological advances will challenge humans to adapt to earth-discrepant gravity conditions. A look at the existing literature shows that learning the particular skill of dealing with gravities other than 1g is highly problematic for the perceptual system. The three main reasons seem to be: (1) our inability to perceive arbitrary accelerations; as such (2) the inherently multimodal nature of gravity perception; and (3) the special characteristics of the internal gravity prior, or in terms of a Bayesian framework, earth gravity’s status as a strong prior.

The present article reviews evidence brought forward in the literature on whether and to what extent humans can adapt to non-earth gravities. On a more theoretical level, it places the internal representation of gravity within a Bayesian framework of perception. For this purpose, several aspects of the topic are reviewed: Section “Gravity Information in Vision Related Processing: What is It Useful and Used for?” provides an overview of the different computations in which gravity is involved, while Section “Attunement to Earth Gravity: Interception Performance under Earth-Discrepant Gravity Conditions” takes a closer look at available data on human interception performance under 0g. Section “Gravity in a Bayesian Framework of Perception” discusses two studies that provide evidence for adaptation to non-earth gravities and uses these as a base for a discussion of an internal model of gravity in a Bayesian framework. It is argued that the reviewed evidence supports envisioning gravity as a strong prior in the Bayesian sense. Finally, in Sections “Conclusions and Further Research”, the discussion is summed up and a few concrete perspectives for further research in gravity perception are given.

## Gravity Information in Vision Related Processing: What Is It Useful and Used For?

To get a better idea of the areas of perception affected by altered gravity conditions, the following part will outline areas of computations for which gravity has been shown to play a role.

### Catching

Since gravity affects the flight of moving objects, one area in which gravity has been given some attention is interceptive timing. Modeling in this area aims at adequately predicting human performance in a variety of catching tasks. Traditionally, the proposed models have strongly relied on information available directly from the optic flow, such as tau (Lee, 1976; Lee and Reddish, 1981) which signals the time to contact of an object under some visual conditions (constant velocity, head-on approach) and consists of combining the visual angle and its rate of expansion. Binocular optic information such as binocular disparity (Rushton and Wann, 1999; Gray and Regan, 2004) can also contribute to extract time-to-contact (TTC) information. As more and more evidence became available for the fact that physical prior information like known size (López-Moliner et al., 2007; López-Moliner and Keil, 2012) or object familiarity (Hosking and Crassini, 2010) seems to be used by the perceptual system to more accurately estimate TTC (that is the remaining time until an object reaches a predefined target such as the observer, a certain point on a screen, etc.), another physical variable came into focus: (earth) gravity. Gravitational biases have been reported for free falling targets (McIntyre et al., 2001; Zago et al., 2005, 2008, 2010, 2011) as well as for parabolic trajectories (Bosco et al., 2012; Diaz et al., 2013; Delle Monache et al., 2014; de la Malla and López-Moliner, 2015; Lacquaniti et al., 2015, validated a gravity based model for parabolic interception brought forward in Gómez and López-Moliner, 2013), objects on ramps (Mijatović et al., 2014), and even for objects that move horizontally (De Sá Teixeira et al., 2013; De Sá Teixeira, 2016). Interestingly, there seem to be certain constraints as to when computations can access the internal representation of gravity; when the so called “idiotropic vector” along the vertical body axis, for example, is not aligned with the direction of gravity, the contribution of the internal representation of gravity decreases (De Sá Teixeira, 2014; De Sá Teixeira and Hecht, 2014). In absence of physical gravity, on the contrary, the use of internally represented gravity information is not necessarily suspended (McIntyre et al., 2001).

In addition to this behavioral evidence, some studies have delivered brain imaging evidence to the case. One study measured brain activation of subjects viewing objects fall downwards with 1g acceleration and compared it to activation when viewing 1g upwards acceleration (that is, −1g). The previously mentioned performance advantages for +1g conditions were here accompanied by differential brain activation in insular cortex, temporoparietal junction, premotor and supplementary motor areas, middle cingulate cortex, postcentral gyrus, posterior thalamus, putamen and medial cerebellum (Indovina et al., 2005). This was replicated by Miller et al. (2008) to account for different variations such as context; and another study added 0g motion to the comparison and found similar differential activations between 1g and 0g and between 1g and −1g (Maffei et al., 2010). Furthermore, a causal role of the temporo-parietal junction was established through TMS: hyperpolarization of this area lead to a significant performance decrease when catching objects governed by 1g, but not for objects governed by −1g (Bosco et al., 2008). Similarly, a lesion study (Maffei et al., 2016) indicated that patients with brain damage in perisylvian areas performed significantly worse than healthy subjects at intercepting targets dropping with 1g, but not for targets moving upwards with 1g acceleration.

### Motor Planning and Control

A second important vein of research at the intersection of gravity and cognition is motor planning and control. Hypogravity and hypergravity affect different motor parameters of catching, grasping and pointing. An increase in variability under earth-discrepant gravity conditions (Bock et al., 1992; Crevecoeur et al., 2010) as well as systematic biases such as pointing undershoot in microgravity and overshoot in hypergravity (Bringoux et al., 2012) points towards the attunement of the perceptuomotor system to earth gravity. Unlike TTC estimation mechanisms, motor commands have proven relatively adaptable to new gravity conditions: (Augurelle et al., 2003) provided evidence for the rapid development of an appropriate and constant grip force for microgravity and hypergravity phases in parabolic flight. Furthermore, the central pattern generator, a mechanism co-responsible for driving rhythmic movements such as walking, modifies issued motor commands for rhythmic arm movements according to the gravitational context (White et al., 2008). While instructions from the experiments partially overruled central pattern generator commands in microgravity, it generally adapted limb oscillations to the resonant frequency, that is the energetically optimal frequency for the gravitational environment. Finally, several studies indicate an optimal integration of gravity and other cues for example for single-joint arm movements (Gaveau et al., 2014) and for vertical pointing movements (Crevecoeur et al., 2009). The use of the internal model seems to be at least partially motivated by the goal of minimizing energy spent on movements (Gaveau et al., 2016). The human motor system hence adapts strongly to its dynamic environment and uses sensory input about gravity to plan movements accordingly.

### Body Orientation

Another set of computations to which gravity contributes crucially is the estimation of body orientation. Astronauts reported that while working on inclined surfaces in a lunar 0.166g environment they were sometimes overcome by the sensation that the ground was actually plain. Other astronauts speak of the so called inversion illusion in which, in zero-gravity, they occasionally have the impression of being turned upside-down with respect to their environment (Lackner and DiZio, 2000). These reports underline the common-sense notion that the direction of gravity is crucial for our perception of orientation and suggest that, when gravity cues grow weaker, the contributions of other sources of information have a higher impact on the final percept.

Apart from direction and strength of gravity, two additional factors influence the sense of verticality: visual cues from the environment, such as the orientation of the insides of a space shuttle, and the so called idiotropic vector which is aligned with the current body orientation (Harris et al., 2012). In the recent past, the contribution of gravity has been investigated to some extent. de Winkel et al. (2012) investigated verticality judgments in hypogravity and found that, under gravities as high as 0.57g, verticality was assigned according to visual cues such as the orientation of the environment, rather than according to the gravitational downwards pull. However, there was huge inter-subject variation: one subject judged verticality under gravities as low as 0.03g according to the direction of gravity instead of their body axis. Interestingly, this threshold correlated with age, with older subjects being less sensitive to the gravitational pull. In hypergravity on the contrary, verticality was consistently judged correctly. These results contrast with Harris et al. (2012) who could not find significant differences in body orientation estimates between 1g and a lunar gravity of 0.166g, while a previous study of the same group reported a significant difference between 1g and 0g (Dyde et al., 2009). However, this study did not report on inter-subject variability, so it is only partially in discordance with the high gravity perception thresholds reported for some of the participants from de Winkel et al. (2012). Whatever the exact gravitational threshold value for verticality judgments may be, the picture drawn by these studies is that gravity bears an important role in how we relate with our physical environment. Especially low gravities just below lunar gravity seem to be problematic, as orientation perception is impaired, but the gravitational downwards pull is still in place. In addition to other factors like the weight of the space suit and limited visual field (Harris et al., 2012), this may be an explanation for why astronauts are prone to falling over when walking or working on the moon surface. Several other sub-areas of body orientation perception have been investigated: participants consistently overestimate roll tilt in centrifuge-induced hypergravity (Clark et al., 2015); both the apparent horizon and the visual straight ahead are reported lower than under 1g conditions (Cian et al., 2014); and the discrepancy between perceived longitudinal axis and actual position was greater in weightlessness during parabolic flights than during control conditions on earth (Clément et al., 2007). Last but not least, gravity may play a fundamental role in multimodal sensory integration as an invariant reference frame (Scotto Di Cesare et al., 2014).

### Gravity as a Mediator between Time and Space: Temporal Judgments and Biological Movement

Furthermore, gravity has been suggested as a mediator between spatial and temporal cues (Lacquaniti et al., 2015): for example, participants judged more precisely the time that elapsed during the gravity-governed free fall of an object than for the same movement in upwards or horizontal directions (Moscatelli and Lacquaniti, 2011). What is more, biological motion is to a large extent dependent on the pendulum-like movements of the organism’s limbs, whose frequency and amplitude are in turn governed by gravity. Biological motion is therefore a good example of gravity being on the interface between time and space, a relationship that is also leveraged by the sensory system for the perception of biological movements. In a task where participants had to place a simulated moving animal in the depth of a given scene, they usually chose the depth that made the animal’s movement congruent with biological movements under earth gravity (Jokisch and Troje, 2003). Although physical differences between motion under moon gravity (0.166g) and speed matched motion under earth gravity (1g) are not very marked, Maffei et al. (2015) reported that observers could easily judge if a movement pattern was governed by moon gravity or by earth gravity. Additionally, certain areas (perisylvian areas, frontal and occipital cortex, hippocampus and putamen) were differentially activated when observing motion governed by earth gravity in comparison to motion governed by lunar gravity. Furthermore, both human and nonhuman vertebrates show a preference for right side up biologically moving stick figures over upside down stick figures shortly after birth (Vallortigara and Regolin, 2007; Simion et al., 2008). Since, conceptually, the difference between right side up and upside down condition can be reduced to gravity congruent and gravity incongruent motion (see Indovina et al., 2005 for a similar argument), a possible conclusion is that humans have an innate, gravity-mediated attention bias for spotting other animals. However, another experimental approach to the issue, namely a looking-time-surprise paradigm, provided evidence that 7 month olds expect dropping objects to accelerate and upwards moving objects to decelerate, while at 5 months they do not (Kim and Spelke, 1992). Simion et al. (2008) attributed this divergence in results to the fact that Kim and Spelke (1992) task involved predictions about moving objects, while their own task involved animate entities, for which babies have been shown to have an attention preference.

### Spatial Inferences and other Cognitive Tasks

Furthermore, gravity effects have been discovered for many tasks involving spatial inferences and spatial reasoning. (Clément et al., 2008) showed that exposure to microgravity leads participants to perceive 3D cubes as taller, thinner and shallower than under earth gravity. In the same fashion, when asked to adjust a 3D cube for it to look “normal”, the result was a shorter, wider and deeper object. Another study (Villard et al., 2005) provided evidence that several optical illusions (such as the Reversed-T, Müller-Lyer, Ponzo and Hering illusions), which rely on the effects of linear perspective, were less likely to occur in 0g than in 1g. Other illusions, that are not connected to linear perspective (such as the Zöllner and Poggendorf illusions), however, were not impacted by microgravity. The authors attribute this difference to the idea that gravity mediates the role of linear perspective in 3D processing. Moreover, while on earth there is usually a preferred interpretation of ambiguous perspective figures (with one being perceived in 70% of the cases), this preference disappears after 3 months in space (with both being perceived about 50% of the time), with earth 70:30 homeostasis being reestablished within a week after reentry (Clément et al., 2015). Moreover, the perceived direction of gravity influenced judgments about the stability of objects that were on the verge of falling off a table (Barnett-Cowan et al., 2011). Finally, also the critical aperture, that is the smallest width of a door just wide enough to pass through, was judged lower in weightlessness during parabolic flights (Bourrelly et al., 2016). The overall evidence presented strongly supports the thesis that bodily felt gravity influences spatial reasoning. Further results suggest that even some other cognitive tasks, such as face recognition, may be impaired, while performance for others, like same-different judgments for abstract objects, are unaffected by microgravity (see Grabherr and Mast, 2010 for more details).

Results from these five fields—catching and TTC estimation, motor control, body orientation estimation, the perception of biological movements and spatial reasoning—suggest that our perceptual apparatus might be attuned to earth gravity even more strongly and in many more ways than previously thought. They also make a compelling case that there is no neat separation between visually and bodily perceived gravities and their computational applications in perceptual processes: primarily visual tasks, such as depth perception, are influenced by bodily gravity cues, and primarily bodily tasks, such as verticality and straight ahead judgments, are influenced by visual gravity cues, a phenomenon upon which Section “Gravity in a Bayesian Framework of Perception” is expanding.

## Attunement to Earth Gravity: Interception Performance Under Earth-Discrepant Gravity Conditions

As shown in Section “Gravity Information in Vision Related Processing: What is It Useful and Used for?”, gravity plays a crucial role in a multitude of cognitive tasks. Given that the whole of human evolution and individual development occurred under the influence of Earth gravity, it seems, *a priori*, highly likely that we are in important ways attuned to its specific and relatively invariable value of about 9.81 m/s^2^. In this section, a closer look is taken at object interception. This area provides some intuitive methods for the investigation of gravity perception, such as measuring the predictive power of the internal model of gravity through partial occlusion of trajectories and its effect on interceptive errors. Performance is considered in four different environments: space, parabolic flights, virtual reality and 2D video projections.

### Space

Experiments conducted in space are the most ecological method in the study of gravity-based perception. Due to the obvious technical difficulties, (McIntyre et al., 2001) remains to the present day the only interception study conducted in space. Early during the Neurolab space shuttle mission—on Day 3, Astronauts performed an interception task with an object that was moving downwards towards their hand with relatively low, constant speeds (that is, governed by 0g) of 0.7, 1.7 and 2.7 m/s. While accuracy of the eventual catch was perfect due to task design (McIntyre et al., 2003), the participants initiated their hand movements consistently earlier with regards to TTC than under 1g conditions on Earth. Moreover, they displayed an abnormal pattern of forearm rotation: after the early onset, they reversed the rotation—potentially because they corrected their TTC expectations through online visual cues—and caught the ball at a lower forearm rotation angle than on earth.

### Parabolic Flight

The only parabolic flight study to test catching performance under non-earth gravity (Senot et al., 2012; see Section “Gravity Information in Vision Related Processing: What is It Useful and Used for?” for more details) found that in the weightlessness phases, subjects expected downwards moving targets to decelerate and upwards moving targets to accelerate. Apart from the conclusions that this experiment allows about the multisensory nature of gravity perception, it is striking that a perceived upwards gravity leads subjects to anticipate a concordant target movement. After all, previous studies indicate that subjects will nearly always use an internal model of earth gravity, that is, they will expect downwards moving objects to accelerate and upwards moving objects to decelerate, and there is contradicting evidence to the fact that this internal model remains intact even when bodily cues to the opposite are present (McIntyre et al., 2001). One possible explanation is that vestibular cues may lead subjects to believe that they are turned upside down, an interpretation which is, however, counter-evidence by the fact that when vestibular and other bodily gravity cues are weak, visual cues about body orientation tend to maintain the upper hand (see Section “Gravity in a Bayesian Framework of Perception”). The more likely explanation is that, as each period of weightlessness is preceded by a period of hypergravity, otolith receptors react to the sudden cease of hypergravity with signals that indicate an upwards gravity, as a consequence of which weightlessness after hypergravity is interpreted as upwards gravity. And in fact, the authors found evidence for corresponding otolith receptor responses (see Section “Adaptation to Catching Under Zero Gravity Conditions” for further details on this study).

### Virtual Reality

3D environments in virtual reality remain, due to their favorable ratio of accessibility and ecologicality, the most important method in visual gravity perception. A series of studies (Zago et al., 2004, 2005; Zago and Lacquaniti, 2005b) established that humans expect objects that move downwards to accelerate with 1g gravity, even when their velocity is constant, as indicated by the observation that TTC for 0g targets was consistently underestimated. In the Punching Task design, participants had to punch a ball dropping behind a screen, whose location was indicated by a projection on the screen, in the moment it reached the lower edge of the screen. The semi-virtuality of the task can be seen as an asset, as it increases engagement of the participant and ecological validity. Further evidence comes from Senot et al. (2005) who found that, when acceleration and direction of the ball were congruent with earth gravity, error rates were lower than in incongruent trials. Interestingly, the overall lowest error rates were recorded for the constant speed condition. Big parts of the literature predict the gravity prior to be tapped in the case of free-falling target interception, which should result in larger interception errors for objects that move downwards with constant velocities. This inconsistency can, however, be explained by the fact that accelerating objects had a higher final velocity than those dropping at constant speed.

Then again, there is the simplest category of 2D tasks based on on-screen presentation. Here, results are seemingly contradictory: some studies report the use of a gravity prior, as for example Mijatović et al. (2014) who found that participants were judging time of arrival correctly for balls rolling down familiar geometrical shapes (an inclined plane) under 1g conditions, but overestimated time of arrival for −1g conditions, that is, when the ball was rolling up the plane. Similarly, Zago et al. (2010) found that performance for targets on a partially occluded, vertical trajectory was consistently better for 1g than for 0g or −1g; likewise, for partially occluded parabolic trajectories, arrival of the ball was spatially and temporally underestimated for 0g, judged correctly for 1g and overestimated for 2g (Bosco et al., 2012). Zago et al. (2004), on the contrary, reported that participants’ performance in TTC estimation for balls dropping on screen was a reasonable fit for the predictions of a first order model for TTC estimation that does not take into account gravity, while a second model including gravity did not match human performance in this task. To unify these results, it has been argued that in 2D presentation, the internal model of gravity is only accessed if pictorial cues are present to put motion into perspective or if parts of the trajectory are occluded (Zago et al., 2009).

### Two Problems with the Existing Corpus of Research

Two caveats, brought on by Baurès et al. (2007), retain some relevance up to the present date. First of all, the study of gravity’s role in interception has often been limited to free falling objects. In the wake of this observation, some recent studies investigated parabolic and other trajectories: trajectories. Bosco et al. (2012) introduced a combination of weightlessness and hypergravity perturbances and occlusions in 1g trajectories and reported earth gravity biases for interception; (Delle Monache et al., 2014) used eye tracking in a similar design and showed predictive effects of an internal presentation of gravity. A paradigm based on an initial parabolic trajectory and several bounces in combination with eye tracking revealed that gaze movements were consistent with earth gravity based predictions of the ball’s position (Diaz et al., 2013). La Scaleia et al. (2015) reported a gravity bias for parabolic interception even when sensory information prior to initiation of the parabolic trajectory indicated an earth-discrepant gravity of 0.2g. And finally, a gravity based model for parabolic interception has been established (Gómez and López-Moliner, 2013) and partially validated (de la Malla and López-Moliner, 2015). Nonetheless, effects of earth-discrepant gravities, especially other than 0g, on naturalistic trajectories have yet to be studied more thoroughly.

Second, when studying an internal model of gravity one also has to take into account the confounding properties of air drag: effectively, it represents a progressive decrease in downward acceleration with increasing velocity with considerable effects on trajectories (d’Avella et al., 2011). While again Gómez and López-Moliner (2013) propose a way of integrating air resistance into their model of parabolic target interception, a relatively accurate and precise gravity prior (as brought forward by authors such as Lacquaniti and Zago) seems contradictory with effectively observed acceleration values of under 9.81 m/s^2^. And in fact, Flavell (2014) suggests that the gravity prior may actually not be set at 1g exactly, but rather at a value between 1g and just below 1g. However, these findings are consistent with an alternative explanation based on the existence of two priors: one relatively inflexible gravity prior and an adaptable air drag prior with information about air resistance and drag coefficients of known objects. This second prior would add to the size prior put forward in López-Moliner et al. (2007). A separate representation of air drag has the additional advantage that it can be employed for both vertical and horizontal movement components, while an integrated gravity and air drag prior is only viable for free fall. While evidence for the existence of a friction prior for movement on surfaces has been presented (Hubbard, 1995; Amorim et al., 2015), the role of air resistance and drag-relevant features of objects in perception remain to be studied more systematically.

Notwithstanding these reservations, many different approaches—from 2D presentation up to experiments conducted in space—have revealed gravitational biases in a big range of computations, which supports the claim that gravity is represented in the brain and accessed in a variety of computations.

## Gravity in A Bayesian Framework of Perception

While Section “Gravity Information in Vision Related Processing: What is It Useful and Used for?” has shown that an estimate of gravity is an important component of many different computations, Section “Attunement to Earth Gravity: Interception Performance Under Earth-Discrepant Gravity Conditions” reviewed evidence that naïve humans perform relatively badly in interception tasks under any condition other than 1g. The question imposing itself at this point is whether humans can adapt to gravities other than earth gravity. To address this issue, the present section first reviews evidence from training studies with 0g stimuli. Then it situates these results within a Bayesian framework and makes a proposal for the place of an internal representation of gravity within this framework.

### Adaptation to Catching Under Zero Gravity Conditions

All the results presented in Section “Attunement to Earth Gravity: Interception Performance Under Earth-Discrepant Gravity Conditions” are based on performance by naive observers, that is, observers that had not received any training at all, nor in the tested gravity, nor in other earth-discrepant gravities. To our knowledge, there are two studies that have investigated to what extent experience in a 0g environment (McIntyre et al., 2001) or explicit training with earth-discrepant, visually perceived gravities (Zago et al., 2004, 2005) can normalize catching in gravities other than 1g. The McIntyre study was mentioned before as the one example in permanent weightlessness in space. In addition to testing in the beginning of the participants’ stay in space, the measures were taken again on the ninth and on the 15th day. No explicit training with the actual task was conducted, but training effects through experience with the general 0g environment can be assumed. And in fact, the result was a naturalization of catching movements: while hand movements, after 3 days just like after 9 and after 15 days, were consistently initiated too early with respect to TTC, they smoothened over time. However, after 15 days they still did not resemble the velocity profile for catching under earth conditions. While the smaller final velocity grants enough time for a slower, smoother interceptive movement, these results still suggest that, at least when both bodily and visual cues indicate a 0g environment, adaptation is possible. Nonetheless, it can be assumed to be a relatively slow process.

Unlike McIntyre, (Zago et al., 2005; Zago and Lacquaniti, 2005a) tested training effects in virtual reality, that is, only visual input served for perceptual adaptation while bodily cues were indicating 1g. They explored whether subjects in their punching design (see Section “Virtual Reality”) could learn to deal with 0g conditions, while at the same time tackling that question whether, in case of positive results, the internal 1g model was adapted or a second internal model for 0g was established. For this purpose, they used different protocols which interleaved 1g and 0g stimuli in different proportions of 0g stimuli (0%, 9%, 50%, 91% and 100%) over one (for the 50% protocol) or two training days (for the other protocols) while on the second day, the proportions of 0g and 1g stimuli was inversed (for example 91% on day 2 for the group that received the 9% protocol on day 1). A first important result is that, regardless of the protocol, performance for 0g improved very quickly, but then, after 5–6 trials, stagnated at an inferior rate in comparison to 1g interception. Also, performance and timing for 1g trials remained constant in every protocol; if the internal 1g model was adapted through training, then performance should worsen for 1g trials: TTC should be overestimated and movement initiation should be delayed. In the same manner, the adoption of a second, 0g model is rather unlikely as movement initiation trended asymptotically towards a value between naïve 0g catching performance and perfect 0g catching performance. Further analysis of the same data indicated that the observed data was, in principle, consistent with an intact 1g gravity representation. Instead the time threshold for movement initiation could have been lowered to the minimum made necessary by processing constraints; due to the uncertainty in the environment, the perceptual apparatus minimizes in this way the timespan for which it has to extrapolate the position of the ball using internal models of physical variables (such as velocity and gravity). And in fact, the plateau value for adaption to 0g interception was 123 m/s in the 50% protocol, which is consistent with previously observed visuo-motor delays for interception (Lacquaniti and Maioli, 1989a; Port et al., 1997).

However, the latter study has not gone uncriticized. Baurès et al. (2007) have pointed out several weak points of the experimental design. The most important disadvantage is a discrepancy in the admissible time window for successfully punching the target between the (virtual) ball displayed on the screen and the actual ball dropping behind the screen in the 0g condition. While both balls crossed the interceptive zone at the same time, they did so with different velocities. In the lowest initial velocity condition (0.7 m/s), the time subjects had to successfully punch the physical target was 24 ms, while the slower motion of the virtual target indicated a time window of 214 m/s. However, as pointed out in Zago et al. (2008), this discrepancy nearly disappeared in the highest initial velocity condition (4.5 m/s), while interception still happened too early. The effect observed by Zago et al. (2004, 2005) can thus not be reduced to different time windows. Baurès et al. (2007) furthermore point out a series of studies (Brenner et al., 1998; Brenner and Smeets, 2005) that suggests that interceptive movements adapt to the velocity of the target, so that different observed velocities during the trajectory influence interceptive movements. Zago et al. (2008) answered this criticism by referring to the fact that movement kinematics (movement speed, peak hand velocity) were constant across different initial velocities (Zago et al., 2005; Zago and Lacquaniti, 2005a). According to their interpretation, these movement parameters are evidence for an attunement to the kinematics of the ball falling behind the screen, thus lending support to the hypothesis of an internal model of gravity.

All in all, discrepancies between onscreen and real ball kinematics in Zago and Lacquaniti’s (2005a) 0g condition are a necessary consequence of the design, which could at least partially account for why participants’ performance in 0g tasks did not improve beyond a certain plateau. They can, however, in no way account for all evidence in favor of in internal model of gravity provided in this study.

### A Bayesian Framework

Perceptual adaptation is generally necessary to deal with new environments and has, as an overarching topic, been treated thoroughly in the literature. One of the most promising frameworks to account for perceptual adaptation is Bayesian perception modeling. This application of Bayesian frameworks originated as a natural answer to the so called Inverse Problem: how does the perceptual system draw conclusions about the actual state of the world from ambiguous and noisy sensory information? This question has been formulated in terms of the concepts of “Encoding” (or “Representation”) and “Decoding” (or “Readout”; Gold and Ding, 2013). The Encoding of the information is what is reported by sensory organs and can be viewed in terms of neural activity in response to a stimulus. Through the process of Decoding, this neural activity is interpreted in order to derive a final percept to guide behavior. There are different strategies for decoding: for example, different sensorial inputs (e.g., information encoded by single neurons or populations of neurons) may be weighted according to their reliability and then combined. The *modus operandi* of Decoding in a Bayesian framework as the integration of* a priori* information, that is, information condensed from the subject’s previous experiences, with sensory information. The weighting of each is inversely related to the variance of the signal, that is, the lower the variance of a signal, the more precise it is, and the higher its weight for the a posteriori guess about the actual state of the world. The following expression is a formalization of this reasoning:

(1) P (world|data)∝P (data/world)×P (world)

which reads as: the probability of the state of the world given the available sensory data (Posterior) corresponds to the probability of the data given a certain state of the world (Likelihood Term) multiplied by the Probability of the world being in a certain state (Prior).

Within this framework, perceptual adaptation can occur in two ways: changes to the likelihood or changes to the prior (Yamamoto et al., 2012). As the prior represents a history of past percepts, it is intuitive how adaptation on the level of the prior takes place: after finishing the processing of each percept, the prior is modified to reflect the difference between the posterior percept and the prior (Miyazaki et al., 2006) through a shift towards the posterior percept. Generally, the human perceptual system aims to minimize this difference, called “prediction error”, in order to prepare for future percepts (Friston, 2009, 2010), which is achieved through the integration of previous percepts in the prior. The adaptation of the prior provides the system with a more accurate representation of statistical properties of the world, allowing it to make more accurate sensory predictions and to minimize the prediction error. This mechanism accounts for attraction effects, that is for situations in which an adaptor shifts subsequent posterior percepts towards the adaptor. But not only the prior allows for changes, but also the likelihood can account for adaptation effects (Stocker and Simoncelli, 2006). In this case, the perceptual system focusses resources to cover the range of the adaptor in order to increase the reliability of sensory input within this range, thus shifting the posterior percept towards the likelihood. It has been suggested that in most cases both opposing mechanisms are at play, with statistical properties of the stimuli favoring one or the other (Yamamoto et al., 2012; Linares et al., 2016).

### The Likelihood: Sensory Gravity Input

In a Bayesian framework, the likelihood function represents the signal provided by sensory organs. Unlike other sensory data such as luminosity or tone pitch, gravity is picked up by different sensory organs: vision, the vestibular system (otolith organs and semicircular canals), proprioception (receptors in muscles and tendons) and other sensory sources (e.g., for somatic cues from kidneys and the intestines); which under normal conditions are subsequently integrated into final percept of the gravity vector.

#### Visual Perception of Gravity

The human visual system has been shown to be rather inept at perceiving accelerations: it seems that acceleration information is not used in order to estimate object movement during occluded parts of a trajectory (Werkhoven et al., 1992; Brouwer et al., 2002; Benguigui et al., 2003; Brenner et al., 2016). How can the visual system thus infer acceleration? The computation of downwards acceleration requires information about the retinal acceleration and about the distance between the observer and the object. Recovering the distance to the target is not always straightforward: a series of cues (eye vergence, accommodation, stereo-disparity, motion parallax; de la Malla et al., 2016) contribute to distance perception, but 2D-presentation or far distances knock out some of these sources of information or make them extremely noisy. However, physical information about the environment (like sizes of familiar objects in the environment) can be used to compute distance. Since these are typically available, distance can usually be computed (Lacquaniti et al., 2014). However, the retinal acceleration as a time derivative of velocity is notoriously hard to estimate (Werkhoven et al., 1992). One hypothesized reason is that, accelerations other than gravity have generally not been relevant to survival during the evolution of the species, so that the skill to perceive them was never developed. All in all, while all the elements necessary for acceleration estimation are in principle available, the visual system does not seem to use them effectively.

#### Vestibular Gravity Cues

The human vestibular system consists mainly of the two otolith organs sacculus and utriculus which serve to measure linear acceleration (forwards and sidewards) and of three semicircular canals which indicate angular acceleration (roll, yaw and pitch). In contrast with the visual system, the vestibular system can detect small accelerations. Notwithstanding this sensitivity, otolith signals are, without further cues, insufficient to differentiate between the gravitational and the inertial components of the gravitational force, that is for example between a forward head tilt and a backwards acceleration. Disambiguation is achieved through a series of “algorithms” (Lacquaniti et al., 2014): (1) longer lasting signals are attributed to head tilts with regards to the gravity vector, while shorter lasting signals are interpreted as linear accelerations (Mayne, 1974). (2) Otolith signals from sacculus and utriculus are combined with semicircular canal signals about the angular velocity of the head (Angelaki et al., 1999; Zupan et al., 2002; Green and Angelaki, 2004). The latter source of information is, however, not always accessible because the semicircular canals only provide information when the head is moving. (3) A solution for steady head situations is the usage of somatogravic feedback, that is, bodily feedback about body orientation with regards to the gravity vector. Additionally, disambiguation mechanisms also rely on an internal model of gravity (Merfeld et al., 1999). More recent contributions have, among other, found single neurons attuned to different head orientations with respect to the direction of gravity (Laurens et al., 2016). For a more detailed discussion of vestibular acceleration and gravity perception, see Lacquaniti et al. (2014).

#### Bodily Gravity Cues

As mentioned before, somatosensory gravity cues can be used to disambiguate vestibular information. However, it is not evident why their role for posterior estimates of gravity should be subordinate to vision or the vestibular system, as a number of studies have shown significant influences of this source of information. For example, pressure working on the insides and outsides of the body have been shown to influence the sense of gravity direction (Trousselard et al., 2004), and the same seems to hold for blood distribution throughout the body (Vaitl et al., 2002). A review on research on the perception of upright in populations with neurological disorders or lesions in vestibular areas confirmed a strong contribution of somatosensory cues (Bronstein, 1999).

Feedback from muscles and other proprioceptive cues are generally taken to be another source of gravity cues (for example Lacquaniti et al., 2014). However, their contribution remains relatively unexplored, with some exceptions such as Barbieri et al. (2008) who showed an influence of proprioceptively perceived gravity on the sense of verticality.

#### Multisensory Integration

Gravity estimates provided by single senses are often noisy or ambiguous. Multisensory integration is therefore useful or even necessary to obtain robust posterior percepts; for example, the use of visual information can disambiguate inconclusive gravitoinertial cues from the vestibular system (MacNeilage et al., 2007). Under normal conditions, vestibular and visual information are combined with other information from muscle and tendon receptors, visceral cues and body orientation (Lacquaniti et al., 2014) for a posterior estimate of the gravity vector. And in fact, it has been shown experimentally that bodily gravity cues influence the visual perception of gravity (Trousselard et al., 2004) and viceversa (especially when bodily gravity cues are weak, as under lunar gravity conditions, Harris et al., 2012). Similar effects have been shown for tasks with more pronounced motor components (Sciutti et al., 2012). The observation that integrative performance across two tasks was highly correlated (De Vrijer et al., 2008) suggests that there is one central mechanism for the integration of different gravity cues.

Another illustrative example for an interaction of vestibular and visual cues is a parabolic flight study conducted by Senot et al. (2012). On parabolic flights, periods of about 20 s of weightlessness are achieved by taking advantage of accelerating an airplane in a specific manner with respect to earth gravity. Importantly, before the actual weightlessness phase, participants experience about 20 s of 1.5–1.8 g hyper-gravity (Karmali and Shelhamer, 2008). Using a combination of parabolic flight and virtual reality, (Senot et al., 2012) had participants perform interception tasks in a 2 × 2 × 2 experimental design of normal gravity/weightlessness, decelerating/accelerating approach of a ball from below/above. Unsurprisingly, participants performed better under normal gravity conditions when the ball was coming from above and accelerating or coming from below and decelerating—as expected for objects governed by earth gravity. For weightlessness, the authors expected the difference to disappear (as a sign of weightlessness being adequately perceived and used for the responsed) or to be maintained as in the normal gravity condition (as a sign of the use of the internal model of earth gravity). However, performance was significantly better for decelerating balls coming from above and accelerating balls coming from below. The authors attributed this to a reaction to the hypergravity experienced immediately before the weightlessness phase. Contrasting with the previously perceived hypergravity (between 1.5g and 1.8g), the consequent weightlessness was perceived as an upwards working gravity. This explanation is supported by the otolith receptor activity observed in weightlessness phases which indeed signaled an upwards acceleration. Further evidence for the inherently multisensory nature of gravity perception comes from an EEG study onboard the International Space Station (Cheron et al., 2014). Significant differences between EEG signals for the perception of 3D images were observed in weightlessness in space, but not under 1g on earth. For a 2D checkered control pattern, however, no EEG differences between weightlessness and earth gravity could be found. And last but not least, Pfeiffer et al. (2013) confirmed the multisensory nature of gravity perception for first-person perspective tasks: both bodily and virtually presented visual directional cues lent contributions to the posterior estimate of the first-person perspective; and De Sá Teixeira (2014) showed contributions of the idiotropic vector to the multisensory aggregate.

It is evident that research on gravity perception must be clear about the inherently multisensory nature of the phenomenon: different sources of information, from visual to vestibular, somatosensory and other cues, interact and inform each other. Their respective weights should depend on the task, with visual cues being more important for interception and vestibular and somatosensory cues prevailing for body orientation tasks, and on the reliability of each estimate. Experimental designs have to take this multimodal nature under consideration in order to isolate exactly those functions they aim to elucidate. Also, and maybe more importantly, it will be necessary to shed light on how exactly different gravity cues are integrated with each other and with the (strong) prior of earth gravity. The success of Bayesian approaches to multisensory integration in areas such as the integration of visual and vestibular cues (Angelaki et al., 2009), as well as Bayesian models accounting for phenomena within the visual and vestibular modalities (Yuille and Kersten, 2006; Laurens and Droulez, 2007) make them, *prima facie*, a useful approach to multisensory integration in gravity perception. However, only very limited attempts have been made so far to place gravity perception processes with a visual focus within a Bayesian framework.

#### Gravity Adaptation on the Level of the Likelihood

Could a change in the likelihood term by selective allocation of resources around the adaptor stimulus allow gravity adaptation to occur? At face value, this mechanism requires a relatively precise sensory representation of the stimulus, as only a distribution with a rather high precision or low variance has a relevant effect on the posterior percept. As indicated by the relatively low thresholds for bodily gravity perception and high thresholds for perception of accelerations of arbitrary direction or strength, only the bodily gravity sensors fulfill this requirement. Furthermore, this kind of adaptation has so far mainly been used to account for effects caused by a short exposure to adaptor and/or effects of short duration (Yamamoto et al., 2012). Finally, the empirical data (McIntyre et al., 2001, 2003; Zago et al., 2005; Zago and Lacquaniti, 2005a) do not support this explanation as gravity adaptation seems to occur in the direction of the adaptor (attraction), while adaptation on the level of the likelihood has been connected to repulsion, that is adaptation away from the adaptor (Linares et al., 2016).

### Gravity as a Strong Prior

**Figure 1:**
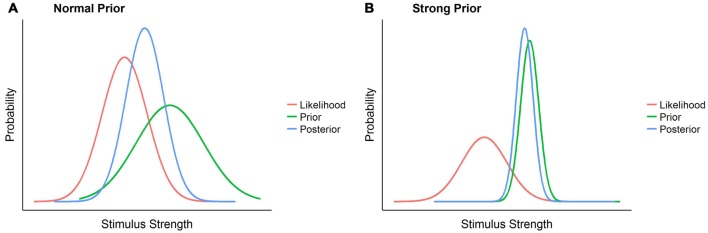
**A comparison between perception in presence of a strong prior and perception in presence of a normal prior.** The normal prior scenario **(A)** represents how likelihood, prior and posterior, represented each as normal distributions, could look like in a normal scenario: a relatively reliable sensory signal, the likelihood, is combined with not particularly pronounced previous knowledge of the world, the prior, and form a posterior percept, with its mean between likelihood and prior and whose variance is lower than that of both. In the strong prior scenario **(B)**, on the contrary, the low variance of the prior attracts the posterior distribution such that the likelihood has an extremely limited influence on the posterior percept, which thus remains very close to the prior.

In the light of the existing empirical data it seems thus much more likely that gravity adaptation occurs at the level of the prior. However, previous studies indicating a slow or asymptotical adaptation do not support the notion of a standard prior that is easily changed with new, contradicting sensory input. While the gravity prior may be relatively plastic or not accessed at all for virtually/2D presented motion (Zago et al., 2004), this doesn’t hold for the more ecological case of real objects or objects presented in an immersive virtual reality environment. Under such circumstances, it resembles the so called strong priors such as the light-from-above or the bigger-is-heavier priors (Adams et al., 2004; Peters et al., 2016). These priors are representations of ubiquitous and persistent properties of the external world, such as the fact that light usually comes from above or that bigger objects are usually heavier, which have been present throughout the whole of humanity’s evolution and every individual human’s development. In Bayesian terms, our lifelong experience with these representations reduces the represented variance of this state of the world to a minimum, making it highly reliable. As a consequence, the weighting of such priors is so high that they easily overrule contrary sensory information represented by the likelihood. Figure 1 provides a visualization of this reasoning.

Nonetheless, evidence has been brought forward that other priors, such as the light-from-above prior or the bigger-is-heavier prior can at least to a certain degree be adapted through experience (Adams et al., 2004; Flanagan et al., 2008). One important difference between these priors and the earth gravity prior is, however, that the necessary sensory information (direction of light and weight/size) is readily available to the perceptual apparatus, while at least the visual system is highly insensitive to accelerations. The second difference is the multimodal nature of sensory gravity input: while the case of conflicts between visually and bodily perceived gravities still needs further empirical investigation, it is warranted to speculate that a full adaptation to gravities other than 1g can only occur when both visual and bodily sensory gravity input indicate the same earth-discrepant gravity value.

### Simulating the Potential Benefits of a Gravity Prior

In the framework outlined above, the role of the internal representation of gravity, or gravity prior, is to aid the decoding process of encoded information by calibrating noisy or ambiguous visual information. In order to illustrate the potential role of assuming a gravity prior in the decoding process of sensory information, we simulate the process of inferring the horizontal velocity component of a parabolic trajectory, which is not directly sensed. This inference is carried out based on two optical variables, the elevation angle (γ) and its temporal rate of change $\left(\dot{\gamma }\right)$. The elevation angle is the angle between the observer’s straight ahead and his line of sight on the object. Its value at time *t* is determined by the following equation:

(2) $$\gamma (t)=tan^{-1}\left(\frac{gt}{2\nu_{h}}\right)$$

with *g* being the underlying gravity and *v_h_* being the horizontal velocity which is, as air drag is neglected for the scope of this simulation, constant throughout the trajectory. Its temporal rate of change is, accordingly, the temporal derivative of this optical variable and indicates how the visual angle changes over the course of a trajectory. Its value at time *t* is determined by the following equation:

(3) $$\gamma (t)=\gamma^{\prime }(t)=\frac{2g\nu_{h}}{g^{2}t^{2}+4\nu_{h}^{2}}$$

**Figure 2:**
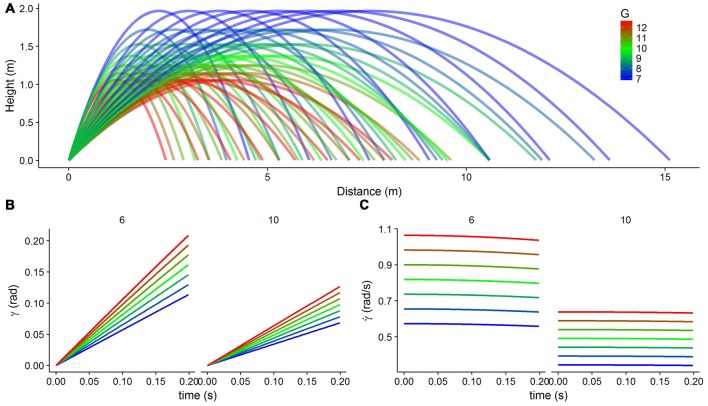
**The physical connection between gravity and the optical variables of elevation angle (γ) and its temporal rate of change $\left(\dot{\gamma }\right)$. (A)** Parabolic trajectories with different horizontal velocities (from 3 m/s to 10 m/s in steps of 1 m/s) and different gravity values (from 0.7g to 1.3g in steps of 0.1g, color coded from blue to red). **(B)** The elevation angle (γ) as a function of time for different gravities (color coded) and two different horizontal velocities (6 m/s, left, and 10 m/s, right), plotted for the first 200 ms of the trajectory. **(C)** The temporal rate of change of the elevation angle $\left(\dot{\gamma }\right)$ as a function of time for different gravities (color coded) and two different horizontal velocities (6 m/s, left, and 10 m/s, right), plotted for the first 200 ms of the trajectory.

**Figure 3:**
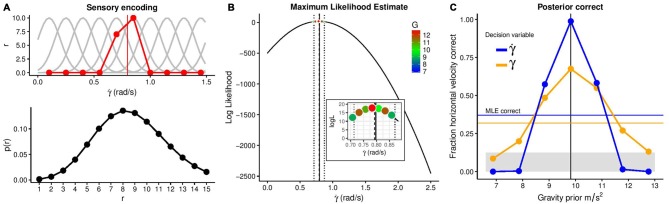
**Illustration of the benefits of a strong gravity prior. (A)** The upper panel shows simulated tuning curves of neurons specialized for different rates of change of the elevation angle (light gray) together with neurons’ responses to the stimulus value of $\dot{\gamma }$ = 0.797 rad/s (red). The lower panel depicts the likelihood of different responses given this stimulation. **(B)** Maximum Likelihood Estimate of the stimulus value given the neural responses with a 10% error margin and the combinations of stimulus values that fall into this error margin. **(C)** Fraction of correct estimates of the horizontal velocity as a function of the gravity prior’s value for a Maximum Likelihood Estimation (thin blue line) and a Bayesian estimation based on the elevation angle (bold yellow line) or its rate of change (bold blue line) as decision variables. The gray area denotes chance level.

Figures 2B,C show the development of these optic variables over the first 200 m/s of a trajectory. Note that the elevation angle (γ) refers to an angular value encoded on the retina of the observer and not to the angle at which the ball is launched relative to the horizontal plane. Note also that the object is approaching the observer frontally on a parabolic trajectory. In order to carry out the simulations, we used 56 different parabolic trajectories (Figure 2A) resulting from combining eight horizontal velocities (from 3 m/s to 10 m/s) and seven gravity values (6.874, 7.856, 8.838, 9.820, 10.802, 11.784 and 12.766 m/s^2^). We defined corresponding tuning curves for γ and $\dot{\gamma }$ that would encompass all possible values of these two optical variables for the time that we considered (0.2 s after motion onset). Figure 3A shows part of the range of the tuning curves (gray solid lines) that we used to encode $\dot{\gamma }$. The covered range was from 0 rad to 1.0 rad for the elevation angle (γ) and from 0.1 rad/s to 2.2 rad/s for its temporal rate of change $\left(\dot{\gamma }\right)$. Within these ranges, there were 11 and 15 peaks for γ and $\dot{\gamma }$, respectively and the standard deviation of the Gaussian curves was 0.1 in both cases.

We simulated 1000 trials in which the system was exposed to 1g and, in any one trial, we randomly selected one trajectory with one horizontal velocity and a gravity of 1g. After encoding γ and $\dot{\gamma }$ for the sampled trajectory, we proceeded to recover the most likely value for the two optical variables (see below). Once the likely values for γ and $\dot{\gamma }$ were estimated through Maximum Likelihood estimate (MLE), we selected all potential trajectories (including all gravities) that matched these values and compared the corresponding selected horizontal velocities with the one used in the encoding to compute the fraction of correct responses. We obtain two possible responses in each trial: one in which we did not take into account the gravity prior with MLE as sole decoding method, and the other in which the MLE was combined with the prior. In order to simulate a strong prior, we used a rather low standard deviation of 0.5 m/s^2^; unfortunately, there is no literature available about the precision with which the gravity prior is represented.

#### Sensory Encoding of Elevation Angle

The first process in the encoding of a sensory representation is to characterize a set of tuning curves for γ and $\dot{\gamma }$ which capture the average response (*r*) of corresponding neuron or detectors depending on the value of these optical values. The red line in Figure 3A (top panel) denotes the activation of the different detectors when a value of $\dot{\gamma }$ of 0.797 rad/s is shown. This value is computed from equation (3) at time *t* = 0.2 s, with a horizontal velocity of 6 m/s and a gravity of 1g. The activation will be different each time the same value of the optical variable is presented which is simulated by adding Poisson noise. The probability of the response (*r*) for a particular value of $\dot{\gamma }$ then is:

(4) $$p(r)=f{\left(\dot{\gamma }\right)}^{r}\frac{e^{-f(\dot{\gamma })}}{r!}$$

where *f* describes the average activity (tuning function). The bottom panel in Figure 3A depicts this distribution, of which the red line in Figure 3A (top) is a particular instantiation. Exactly the same process can be carried out using γ as an optical variable. In each of the 1000 trials, this process was conducted and the horizontal velocity was changed on a trial-to-trial basis.

#### Decoding of the Rate of Change of the Elevation Angle

During Decoding, for a pattern of response (*r*) like the example shown in Figure 3A (top), the system infers the “true” value of the stimulation, in this case the value of the elevation angle at this particular time (0.2 s) or the value of its temporal derivative. A very common rule, without considering any prior information, is to use the MLE:

(5) $$L(\dot{\gamma }|r)=p(r|\dot{\gamma })$$

Here, solely the information of the very same encoding is used to infer the value of $\dot{\gamma }$. Figure 3B shows the estimated MLE for the same value as in Figure 3A $(\dot{\gamma }$ = 0.797). As very similar values of $\dot{\gamma }$ can correspond to different trajectories with different gravities or horizontal velocities (recall the stimulus in this example had a horizontal velocity of 6 m/s and 1g), the inset in Figure 3B shows possible stimuli within a range of 10% about the MLE estimation of $\dot{\gamma }$. Also, note that a MLE rule for decoding would recover a higher value of horizontal velocity and gravity (red dot in the inset of Figure 3B). The very same process was simulated in 1000 trials. In each trial, a different trajectory was encoded and decoded, with horizontal velocities ranging from 3 m/s to 10 m/s (in increments of 1 m/s) and a fixed gravity value of 1g. Figure 3C shows the proportion of times that the correct horizontal velocity is recovered when assuming different gravity values. Additionally, the proportion of correct responses based on the MLE (without any gravity prior) is displayed. The figure illustrates how the use of a strong (terrestrial) prior dramatically increases the proportion of correct responses. As a second decision variable based on which the horizontal velocity could be recovered, we use the elevation angle (γ), simulating the same process. Due to its higher ambiguity with regards to the horizontal velocity, this results in a lower proportion of correct responses. We conclude that the optical variable corresponding to the rate of change of the elevation angle is the one whose use as a decision variable benefits the most from the application of a prior knowledge of gravity. Arguably, one can regard the high fraction of correct responses due to the use of the correct prior as rather trivial. However, we want to showcase that the high degree of ambiguity conveyed by optical variables can only be circumvented by assuming the correct prior. Note that MLE performs much more poorly due to the inherent ambiguity of the optical variables (i.e., similar encoded values are consistent with different physical parameters, such as horizontal and vertical velocities, and even different gravities). Of course, assuming the wrong prior distribution of gravity values leads to systematic errors (McIntyre et al., 2001) and adaptation to the new environment is necessary to correct them.

As a final remark, the present simulation is intended to show a simple example in which the use of a gravity prior can enhance the decoding of ambiguous optical variables. Of course, real life encoding/decoding is much more complex, as more sources of information is available to recover relevant physical variables for perception and action. For example, optical variables related to looming information whose encoding/decoding can play a role especially in the later part of the trajectory (Gómez and López-Moliner, 2013; de la Malla and López-Moliner, 2015) convey additional ambiguous information. For the sake of simplicity, however, we forewent additional optical variables and focused on the elevation angle and its temporal rate of change.

## Conclusions and Further Research

While further, consolidated experimental data is required, the existing corpus of research gives rise to some preliminary conclusions: it is useful to envision the gravity representation in the human brain as a strong prior in the Bayesian sense. As such, it may aid human perception by calibrating ambiguous or unreliable visual information from other sources such as optic flow. Hence, our hypothesis unifies results on human catching performance under noisy visual conditions on earth, performance under earth-discrepant gravity conditions and adaptation to non-earth gravities within a predictive coding framework.

While some findings in mice indicate that gravity conditions during development may actually not be crucial for gravity perception in later stages of their lives (Beraneck et al., 2012), it is still reasonable to believe that Earth gravity’s consistent presence during human evolution and development, as well as the inability of the visual system to pick up accelerations as such, are serious limitations to an adaptation of this prior after experience with gravities other than 1g. Especially when only visual cues indicate an Earth-discrepant gravity, while bodily cues (vestibular, intestinal and muscle/tendon responses) signal Earth gravity, the gravity prior remains largely intact (Zago and Lacquaniti, 2005a). However, methodological issues as well as possible alternative explanations that do not involve changes to the gravity prior spotlight the need for further research in the area.

Further research could focus on evidentiating the nature of the internal model of gravity in a Bayesian framework. To this end, it would be beneficial to expand on previous adaptation studies (McIntyre et al., 2001, 2003; Zago et al., 2004, 2005); especially the respective contributions of gravity’s status as a strong prior and its multimodal nature to the limited adaptation to non-earth gravities remain to be disentangled. To this end, further adaptation studies with manipulations of the different gravity cues will have to be conducted. As the timing restrictions of parabolic flights pose serious limitations to a potentially slow and effortful adaptation, testing in space or on the surface of the Moon or Mars is certainly a long-term goal for this line of research. Second, it remains to be studied why adaptation in tasks with a pronounced motor component happens comparably fast (for example Crevecoeur et al., 2009; Gaveau et al., 2011), but extremely slow for catching and TTC estimation. One hypothesized reason is that the latter relies more on predictive mechanisms than the former. Last but not least, the study of clinical populations could prove advantageous: if the internal model of gravity is indeed to be considered a prior and the Weak Prior Hypothesis for Autism (Pellicano and Burr, 2012) or similar theories (Lawson et al., 2014; Van de Cruys et al., 2017) hold, persons with ASD should perform better at interception tasks under earth-discrepant gravity conditions. Likewise, Schizophrenia has been linked to abnormalities in the integration of sensory input and prior beliefs and prediction errors (Fletcher and Frith, 2009). Strong priors (e.g., gravity) could thus be employed to tease out the mechanisms underlying these conditions and viceversa.

## Author Contributions

BJ performed literature research starting from JL-M’s suggestions; JL-M programmed the simulations and provided general guidance; BJ and JL-M wrote the article.

## Funding

The first author was supported by the following grant: Ajuts per a la contractació de personal investigador novell (FI-2016). Funding was provided by the Catalan government (2014SGR-79) and Ministry of Economy and Competition of the Spanish government (PSI2013-41568-P). The second author, JL-M, was supported by an Institució Catalana de Recerca i Estudis Avançats Academia Distinguished Professorship award.

## Conflict of Interest Statement

The authors declare that the research was conducted in the absence of any commercial or financial relationships that could be construed as a potential conflict of interest.
